# A Rare Ectopic Localization of Hidradenoma Papilliferum in the External Auditory Canal

**DOI:** 10.7759/cureus.91457

**Published:** 2025-09-02

**Authors:** Riham Altaisan, Nikolaos Zirganos, Joackim Mahdjoub, Laurent Tavernier

**Affiliations:** 1 Department of Otorhinolaryngology, Head and Neck Surgery, King Faisal University, Al-Ahsa, SAU; 2 Department of Otorhinolaryngology, Head and Neck Surgery, Centre Hospitalier Universitaire (CHU) Besançon, Besançon, FRA; 3 Department of Cellular and Anatomical Pathology, Centre Hospitalier Universitaire (CHU) Besançon, Besançon, FRA; 4 Department of Otorhinolaryngology, Head and Neck Surgery, Université Marie-et-Louis-Pasteur, Besançon, FRA

**Keywords:** adnexal neoplasm, apocrine tumor, case report, ceruminoma, ent, external ear, hidradenoma papilliferum

## Abstract

We describe the case of a 57-year-old man with a 2-year history of intermittent left-sided ear fullness and recurrent cerumen impaction. Otoscopic examination revealed a soft, flesh-colored pedunculated mass on the posterior wall of the left external auditory canal (EAC). Temporal bone computed tomography demonstrated a 6 mm pseudonodular lesion with minimal underlying osteolysis. The patient underwent surgical excision under local anesthesia, and histopathological evaluation confirmed the diagnosis of hidradenoma papilliferum (HP) with no evidence of malignancy. This case highlights the importance of considering HP in the differential diagnosis of EAC tumors, as its presentation is often non-specific. Radiological assessment, complete surgical excision, and histopathological analysis remain essential for accurate diagnosis and effective management.

## Introduction

Hidradenoma papilliferum (HP) is a rare benign adnexal tumor that typically arises in the anogenital region in women and is thought to originate from apocrine glands under hormonal influence, as these lesions often express estrogen and progesterone receptors [1]. Clinically, HP presents as a well-circumscribed, slow-growing, cystic papule that is usually asymptomatic, though ulceration or malignant transformation has been reported in rare cases [1].

Ectopic presentations of HP are very rare but have been described in areas containing modified apocrine structures, particularly in the head and neck region, including the ceruminous glands of the external auditory canal (EAC) [1,2]. These glands, located deep in the skin of the cartilaginous portion of the EAC, are modified sweat glands responsible for cerumen production [2,3]. In these cases, patients typically present with unilateral conductive hearing loss, occasionally accompanied by otalgia or otorrhea due to secondary otitis externa from canal obstruction [2-4]. However, in our case, the patient presented with different symptoms that have not been previously reported in the literature.

Given the rarity of HP in the EAC and its potential for diagnostic challenges, we report a new ectopic case to expand the limited literature and emphasize the need to consider this unusual localization. To the best of our knowledge, only four cases of HP localized in the EAC have been reported in the English literature since 1981 [1-4].

## Case presentation

Clinical presentation

A 57-year-old male with no significant medical or surgical history presented with a 2-year history of intermittent left-sided ear fullness, frequently associated with recurrent cerumen impaction. He denied otalgia, otorrhea, or tinnitus. Otoscopic examination revealed a soft, smooth, flesh-colored pedunculated mass arising from the posterior wall of the left EAC, partially obstructing the canal. Audiometry demonstrated normal bilateral hearing thresholds.

**Figure 1:**
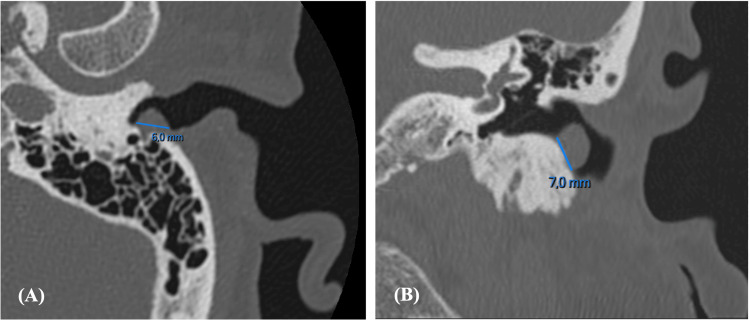
Left temporal bone CT demonstrating a pseudonodular cutaneomucosal tumor of the external auditory canal in (A) axial and (B) coronal views

Temporal bone computed tomography (CT) showed pseudonodular cutaneomucosal thickening measuring 6 mm at the entrance of the left EAC, associated with minimal underlying osteolysis of approximately 2 mm (Figure 1). The tympanic membrane appeared intact, with no abnormalities. Given the persistence of symptoms and the imaging findings, surgical excision under local anesthesia was recommended. 

Surgical intervention

The procedure was performed through a transcanal approach under local anesthesia. A Beaver blade was used to make an incision anterior to the lesion. Serous fluid was aspirated from within the mass and submitted for bacteriological analysis to rule out any underlying infection. The lesion was then meticulously dissected down to its base and completely excised. During surgery, a small erosion of the EAC bone underlying the lesion was observed. The surgical specimen was subsequently submitted for histopathological examination. To reconstruct the defect, a perichondrial graft was harvested from the retroauricular conchal cartilage and applied to the EAC.

Follow-up and outcomes

Postoperative recovery was uneventful. At one-month follow-up, the patient reported complete resolution of symptoms, and clinical examination showed complete local healing. At the three-month follow-up, no recurrence was observed.

**Figure 2:**
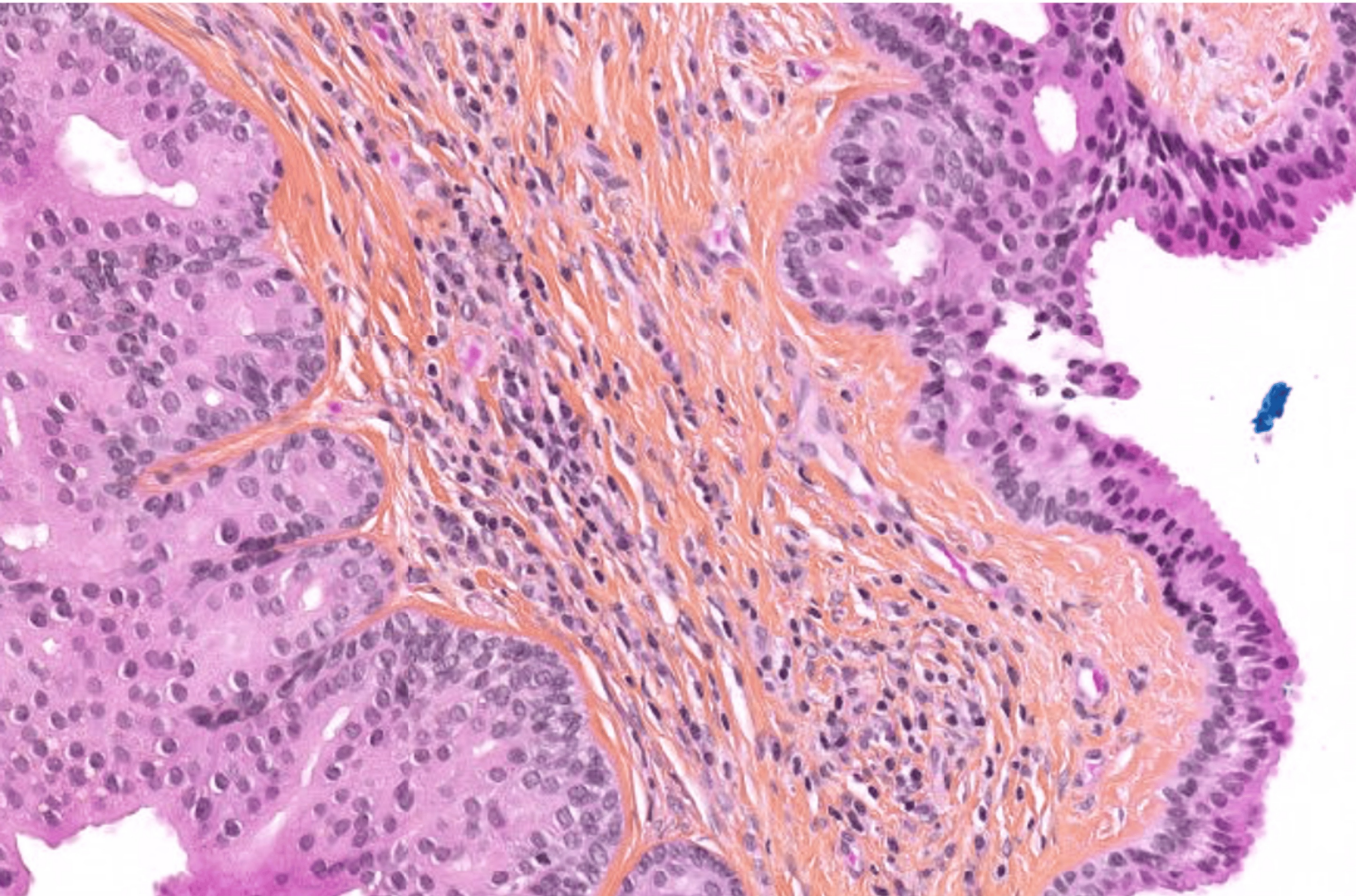
Hidradenoma papilliferum: composed of two distinct epithelial layers, with basal cuboidal cells underlying a luminal layer of columnar cells

Final histopathological analysis confirmed a well-circumscribed nodule measuring 6 × 7 mm, composed of papillary and glandular structures lined by a double epithelial layer - luminal columnar and basal cuboidal cells - consistent with HP (Figure 2). There was no evidence of hyperplasia or any features suggestive of malignancy. Bacteriological cultures of the aspirated fluid were negative.

## Discussion

Tumors of the ceruminous glands, historically classified under the outdated term “ceruminomas,” represent a heterogeneous group of neoplasms with diverse biological behavior [4]. They account for approximately 5% of all tumors of the external ear [5] and encompass a wide spectrum, ranging from benign lesions, such as adenomas, pleomorphic adenomas, and syringocystadenomas, to malignant forms, including adenoid cystic carcinoma, mucoepidermoid carcinoma, and adenocarcinoma [2,4]. Among the benign variants, HP remains exceptionally rare, particularly when arising outside its classic anogenital location. The presence of HP in the EAC reflects the shared secretory properties of ceruminous glands, which exhibit both apocrine and eccrine differentiation [2].

Histologically, HP is characterized by cystic eosinophilic spaces associated with papillary and adenomatous structures lined with a double epithelial layer: an inner luminal columnar layer and an outer basal cuboidal layer, considered the hallmark of apocrine differentiation [1,2]. Immunohistochemistry, though not always required, can support the diagnosis using markers such as epithelial membrane antigen, carcinoembryonic antigen, gross cystic disease fluid protein-15, and human milk fat globule membrane antigen [1,6]. The main histologic differentials include tubular apocrine adenoma, clear cell hidradenoma, and syringocystadenoma papilliferum [7].

Clinically, HP in the EAC has no pathognomonic features distinguishing it from other tumors of the ear canal [3]. Across the reported cases of HP of the EAC, patients presented with a wide age range (32 to 78 years) and included both men and women [1-4]. Presentations are often non-specific. The most common presenting symptoms described in the literature were conductive hearing loss, otalgia, and otorrhea, although one recent case described a long-standing papule with recurrent fluid drainage (Table 1) [1-4]. In our case, the patient reported different symptoms, which are intermittent aural fullness and recurrent cerumen impaction, correlating with a pseudonodular thickening at the entrance of the EAC. Given this non-specific presentation, imaging plays a crucial role in preoperative assessment. Computed tomography is particularly useful for evaluating local extension, excluding bone invasion, and assisting in surgical planning [2,5]. In our case, CT revealed limited osteolysis of the posterior EAC wall, which raised the concern for more aggressive pathology and prompted surgical excision. To our knowledge, none of the previously reported cases of HP of the EAC have described underlying osteolysis, which is an atypical finding for such a benign lesion [2,3]. Histopathology, however, remains the gold standard for a definitive diagnosis [1,3].

**Table 1:** Summary of reported cases of hidradenoma papilliferum of the external auditory canal EAC: External auditory canal, CT: Computed tomography, GA: General anesthesia

Author / Year	Age / Gender	Presentation & Symptom Duration	Lesion Size	Investigations	Management	Follow-up
Shimon SV, et al. 2024	43-year-old female	Long-standing papule in the right conchal bowl of the EAC with recurrent fluid drainage for >5 years	Not specified	Shave biopsy	Surgical excision	Not reported
Laababsi R, et al. 2020	56-year-old male	Right ear hearing loss for 5 months	1.5 cm	Biopsy; temporal bone CT	Surgical excision under GA via combined retroauricular and endaural approach	3-month follow-up: no recurrence
Drvis P, et al. 2012	78-year-old female	Right ear hearing loss, otalgia, and chronic otorrhea for 1 year	6 cm	Biopsy; temporal bone CT	Surgical excision under GA via combined retroauricular–endaural approach; second-stage EAC reconstruction with regional skin flap	1-year follow-up: no recurrence
Nissim F, et al. 1981	32-year-old female	Left ear obstruction and hearing loss for 6 months	1 cm	Not specified	Surgical excision under GA via retroauricular approach	1-year follow-up: no recurrence

Surgical excision with clear margins is considered the treatment of choice and is typically curative for benign HP [1,2]. While most reported cases, including ours, achieved good outcomes with single-stage excision, other authors have described staged approaches with delayed reconstruction until histological confirmation is obtained [3]. Although malignant transformation of HP is exceedingly rare, cases of intraductal carcinoma and invasive adenosquamous carcinoma arising in association with HP have been reported [1,3]. In such cases, extended resection or adjuvant radiotherapy may be necessary [2]. This highlights the importance of thorough excision and close follow-up, especially when lesions present atypically. Interestingly, some authors have speculated about a possible association between human papillomavirus (HPV) infection and malignant evolution of HP, though this link remains unproven [8].

## Conclusions

This case underscores the importance of including rare apocrine tumors, such as hidradenoma papilliferum (HP), in the differential diagnosis of EAC lesions. Due to their non-specific clinical presentation, these tumors can easily mimic more common benign or malignant masses. Radiological assessment combined with surgical excision and histopathological evaluation remains essential for accurate diagnosis and effective management. Although HP is benign, close follow-up is recommended, particularly in cases with close or incomplete margins, to monitor for potential recurrence or rare malignant transformation.
